# Analysis of Injuries in the Swiss U20 Elite Ice Hockey Season 2019/2020—A Retrospective Survey

**DOI:** 10.3390/sports12040088

**Published:** 2024-03-25

**Authors:** Jonas Ruff, Jan Taeymans, Angela Blasimann, Slavko Rogan

**Affiliations:** 1 Division of Physiotherapy, School of Health Professions, Bern University of Applied Sciences, Murtenstrasse 10, 3008 Bern, Switzerland; jonas.ruff@students.bfh.ch (J.R.); jan.taeymans@bfh.ch (J.T.); slavko.rogan@bfh.ch (S.R.); 2 Department of Movement and Sports Sciences, Faculty of Physical Education and Physical Therapy, Free University of Brussels, Pleinlaan 2, 1050 Brussels, Belgium

**Keywords:** ice hockey, wounds and injury, incidence, prevalence

## Abstract

(1) Background: In Switzerland, there is little data on injury characteristics in elite ice hockey players aged under 20 years (U20 Elite juniors). This study aimed to determine the injury rate and type of injury in Swiss U20 ice hockey players. (2) Methods: The present study was carried out in a retrospective, non-experimental design using an online questionnaire provided to the 314 elite players of the 12 Swiss U20 Elite ice hockey teams. The injury rate, rate ratios, injury location, type and severity of injury, and injury mechanism were reported. (3) Results: Seventy-three athletes from 11 teams volunteered (response rate = 24%). A total of 30 out of 45 recorded injuries led to time loss in practice and competition. Injury occurred once or twice during the 2019/2020 season. For each player, the injury rate was 0.66 per 1000 practice hours and 2.98 per 1000 competition hours (injury rate ratio = 4.5). The head/neck region was the most common injury location (45.5%). (4) Conclusions: Knowledge of injury characteristics in ice hockey is necessary for meaningful injury management and injury prevention. The results of the present study provide information on the injury rate, location, types, severity, and mechanism in elite Swiss U20 ice hockey players. Most injuries result from contact with another player. More strict sanctioning for irregular behavior and fair play can serve as preventive measures. Further studies should examine different preventive measures such as wearing full-face coverage.

## 1. Introduction

Ice hockey is the fastest team sport [1,2], played on thin steel blades [3], and is characterized by high-intensity intermittent skating with an ice hockey stick, a fast-moving puck, rapid changes in velocity, and frequent physical body contact [4]. Injuries range from minor conditions (e.g., superficial wounds) to more severe problems (e.g., traumatic brain injuries). Fatigue, excessive body contact by the opponent, or collision with boards have been described as conditions in which players are highly prone to injuries [5,6,7,8,9]. Aggressiveness, age and environmental conditions, equipment, general skills, lacking the constitution for ice hockey, nutritional status, physical and mental fitness, sports equipment, and technique are also important risk factors for injury in ice hockey players [10].

Ice hockey injuries result in time loss from practice and games, and may lead to a high economic burden on society [11]. Between 2002 and 2006, the injury incidence rate in Switzerland was 1400 injuries per million hours of playing ice hockey, but this number increased to 2017 injuries per million hours of playing between 2009 and 2013 [12,13].

The differentiation and interpretation of sports injury rates are pertinent to the role of athletics trainers, coaches, and medical staff, which are partly responsible for promoting the safety of sport in society [14]. Therefore, an in-depth understanding of the epidemiology, etiology, and risk factors of sports injuries is of utmost importance. Athletics trainers, coaches, and medical staff should be able to accurately interpret and apply injury data and statistics to recommend medical resources, such as more staff, better services, safer facilities, and equipment. If they understand these approaches well, they will be able to make safer return-to-play decisions and reduce the likelihood of re-injury in ice hockey athletes.

While epidemiological data are well documented in professional ice hockey, there is a lack of such data in Swiss U20 ice hockey. An overview of the most common injury sites, the injury severity, and the associated practice sessions and games missed can help players, coaches, and medical staff to make more informed return-to-play decisions. In addition, such data are needed to address adequate diagnostic tools, optimize treatment modalities, and plan and implement prevention interventions to avoid injuries in Swiss elite U20 ice hockey players.

### Aim

The aim of this study was to assess the injury rate, injury rate ratio, injury locations, injury types, as well as injury severity and injury mechanism in Swiss U20 ice hockey players during the 2019/2020 season.

## 2. Materials and Methods

### 2.1. Design

This retrospective quantitative study was conducted using an online questionnaire survey based on the validated questionnaire by Theisen et al. [15] and the Injury Report Form (IRF) of the International Ice Hockey Federation (IIHF). The questionnaires collected anthropometric characteristics (age, body weight, height), weekly practice and competition volume (seasonal: peak season, off-season), date of injury, body parts involved, mechanism of injury, injury frequency, injury type, injury severity, and other injury-related details from Swiss U20 elite ice hockey players during the 2019/2020 season.

The questionnaire was presented in German, French, Italian, and English to avoid errors due to language comprehension. Translated versions were checked several times by independent experts for language errors and comprehensibility and were adapted if needed. Questionnaires were filled out in March 2020. Coaches of teams with a low response rate were contacted once to ask them to remind their athletes to volunteer.

The present study followed up on previous studies by Taeymans et al. [2] and Engel et al. [16] with the common objective of analyzing sports injuries in team sports such as ice hockey or floorball to establish predictors of injury in those team sports.

To ensure that the questionnaire accurately captured the nature of injuries and their influencing factors, the following questions were added to the questionnaires: (a) What was the extent of practice and competition for Swiss U20 ice hockey during the 2019/2020 season? (b) What was the incidence of injury among Swiss U20 ice hockey players during the 2019/2020 season? (c) In what context did Swiss U20 ice hockey players sustain injuries? (d) What were the causes of the injuries? (e) What types of injuries occurred? (f) How much time did Swiss U20 ice hockey players miss sport participation (training and competition) due to injury?

### 2.2. Recruitment of the Participants

This study has pilot characteristics because previous research in Switzerland was related to amateur and not junior U20 ice hockey players at the highest level. The coaches of all 12 Swiss U20 ice hockey teams were informed by email about the aims of this study. Coaches provided the online questionnaires to all 314 U20 Swiss ice hockey players of the 12 teams competing in the Swiss U20 ice hockey league in March 2020. Questionnaires with missing data were excluded from the analysis. Volunteers received written information about the academic aims of this study and provided written consent. The chosen method ensured the anonymity of players and teams. This retrospective pilot study was approved by the Ethics Committee of Canton Bern (KEK-BE: 2017-00236).

### 2.3. In- and Exclusion Criteria

Ice hockey players born between 2000 and 2003 who had played in one of the 12 Swiss U20 ice hockey teams during the 2019/2020 season were eligible. All injuries leading to time loss in at least one practice session or ice hockey match, and which occurred during a practice session or a match were relevant for the present investigation. Exclusion criteria for the data analysis were incomplete questionnaires and reported injuries that occurred outside of practice or the game schedule of the Swiss U20 ice hockey championship.

### 2.4. Definitions

In this study, “sports injury” was defined as follows: a lesion that occurred during practice or competition and that resulted in the ice hockey player’s inability to participate in a subsequent practice session or ice hockey match [17].

Time loss from a practice or game is often used as an indicator for injury severity. Therefore, the number of practice and game days missed due to injury was included in the questionnaire [1,18,19]. The time loss due to the reported injuries was categorized as “less than one week”, “between one and two weeks”, “between two and four weeks”, and “four weeks and more”. Four weeks of time loss was defined as the cut-off to distinguish between “minor” and “severe” injury.

Injury mechanism was defined as the manner in which the player sustained the injury [2,16]. In other words, it refers to the way by which trauma and its associated forces (in)directly damage the athlete’s body (e.g., skin, muscles, organs, and bones).

Athletic exposure (AE) was illustrated as a component of injury rate (IR) [20] and has been defined as one athlete participating in one practice or game in which there is a potential for athletic injury [21].

IR was defined as the number of injuries per 1000 h of exposure playing ice hockey [16], as injuries per 1000 practice hours, as injuries per 1000 game hours, or as overall injuries per 1000 AEs (practices and games combined). This method is considered a more accurate measure to define a sports injury [22].

### 2.5. Data Extraction

Two independent researchers transferred the data from the online survey tool SurveyMonkey (SurveyMonkey Inc. Location: San Mateo, CA, USA) into an electronic spreadsheet (Microsoft Excel, 2016). A comparison of the data was carried out afterwards. Any inconsistencies were corrected by consensus. This procedure helped to eliminate errors and to improve data quality [16].

### 2.6. Statistics

The collected data were analyzed by means of the software R (version 4.1.3) [23]. The calculation of athletic exposure metrics such as the IR for practice, game, and whole exposure period was performed as follows: number of injuries divided by the total exposure volume (in hours), multiplied by a factor of 1000. The injury rate ratio (IRR) [1] was calculated by dividing the frequency of game injuries by the frequency of practice injuries [16].

## 3. Results

**Figure 1:**
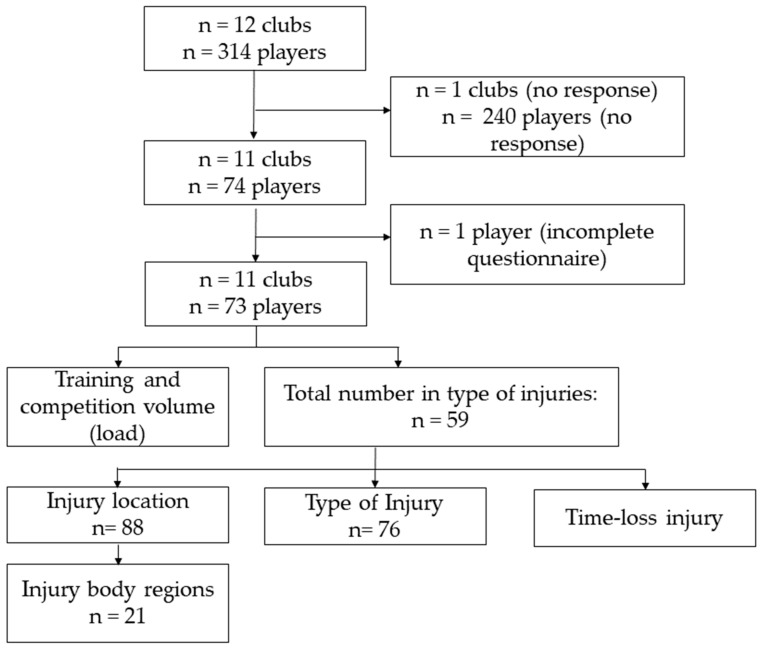
Study flow.

In total, 74 out of the 314 (23.6%) eligible Swiss U20 elite ice hockey players, adhering to 11 of the 12 Swiss U20 ice hockey teams, volunteered in this online survey. One questionnaire was filled out incompletely and was omitted from the analysis (Figure 1).

### 3.1. Participants’ Characteristics

Volunteers’ (*n* = 73) mean body weight and body height were 76.6 ± 6.7 kg and 179.0 ± 5.0 cm, respectively. The distribution of playing positions included eight goalkeepers (11%), 26 defensemen (36%), and 39 forwards (53%). Table 1 illustrates anthropometric data of the Swiss U20 Elite ice hockey players included in this present study stratified for player position.

The observed practice and competition volumes were 599 ± 140 h and 138 ± 37 h, respectively. Table 2 provides an overview of the average exposure volume in a practice and game.

### 3.2. Injury Data

A total of 45 out of 73 Swiss U20 elite ice hockey players (62%) were injured during the 2019/2020 season, resulting in 59 injuries. A total of 28 of the 73 ice hockey players (38%) remained injury-free, while 32 of the 73 volunteering athletes (1.4%) suffered three injuries. More than half of all injuries (51%) occurred during a game. A fraction of 34% of the injuries occurred during practice on the ice and 15% during off-ice practice. The IR in this present sample under evaluation was 0.66 per 1000 practice hours and 2.98 per 1000 game hours. The IRR was 4.5 times higher in-game as compared to practice.

### 3.3. Injury Locations, Types, and Severity

**Figure 2:**
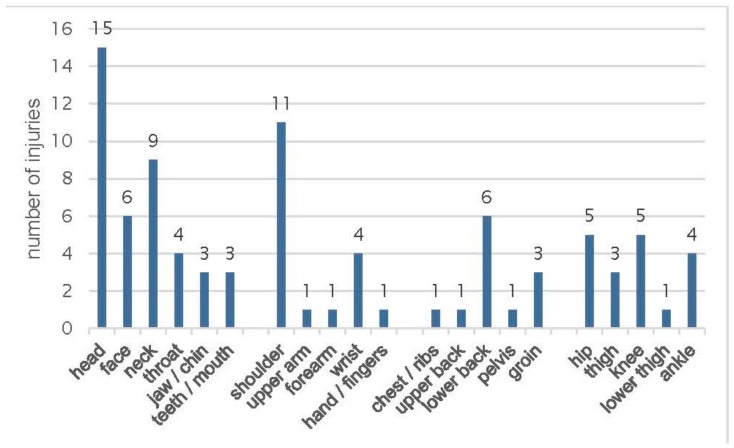
Distribution of injuries by body part injury location.

Athletes volunteering in this study reported 59 injuries classified with 88 injury localizations in 21 body regions (Figure 2). The most often reported injured body region concerned the head/neck (n = 40; 46%). The lower extremities were affected in 18 out of 59 (21%) injuries, representing injuries of the knee (n = 5) and hip (n = 5). A total of 76 different types of injuries were registered. Twelve players reported more than one type of injury. The most common injuries affected the muscles and tendons (20%) as well as contusions/sprains and concussions (18%). Figure 2 shows an overview of the observed injury locations in this sample of 73 junior ice hockey athletes.

**Figure 3:**
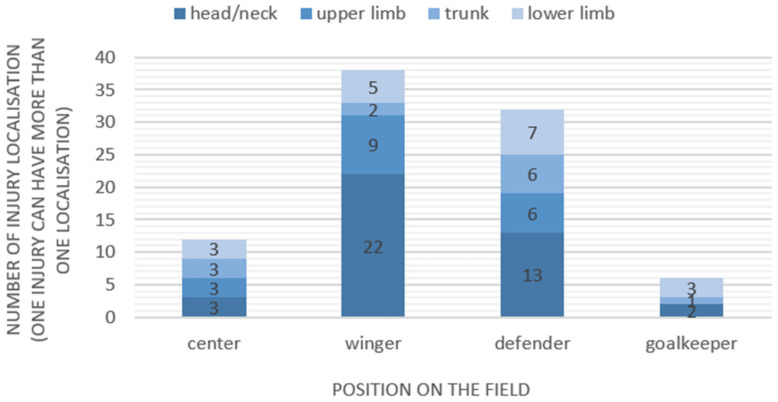
Injury location by player position.

The highest amount of injured body location was reported by wingers (n = 38) where the head/neck was affected in 57.9% (n = 22). In defenders, the head/neck region was also the most reported injured body location, accounting for 40.1% (n = 13) of all affected body regions. Goalkeepers reported no injuries of the upper limb (Figure 3).

In 42% of the injured athletes, the time loss was more than four weeks. The time loss after suffering from a concussion was one week to four weeks (34.5%) and more than four weeks (12.5%). After a fracture or a joint injury, ice hockey players usually had to take a break of more than four weeks. Skin injuries resulted in a time loss of less than one week in 80% of cases. Table 3 illustrates the type of injury in relation to the injury severity.

### 3.4. Injury Mechanism

In 58% of cases, injuries resulted from an external impact (forces acting on the body). Table 4 depicts the injury mechanism in this study as % of injuries.

Around 11.9% of the injuries (n = 7) occurred during the first period, 38% (n = 11) during the second period, and 18.6% (n = 11) during the third period of a game. In addition, 38% of each injury occurred in the last two thirds, 56% of all injuries occurred during one-on-ones, and 28% of all injuries occurred in the player zone.

## 4. Discussion

To the best of the authors’ knowledge, this was the first study in Switzerland that aimed to assess the injury rate, injury rate ratio, injury locations, injury types, as well as injury severity and injury mechanism in Swiss U20 ice hockey players during the 2019/2020 season.

Of the 73 participating elite junior athletes, 45 (62%) reported 1 to 3 injuries (totaling 59 injuries) during this period under evaluation. An injury rate per player of 0.66 injuries per 1000 exposure hours in practice and 2.98 injuries per 1000 exposure hours in games was found. The most common injury location was the head/neck region while muscle and tendon injuries (20%) were documented as the most common type of injury. The time loss, a proxy of injury severity, was more than four weeks in 42% of the injuries. In competition, game duration (38% each of injuries in the last two thirds) and one-on-ones (56% of all injuries), as well as the player zone (with 28% of all injuries), all count as contributing factors to an injury.

Despite contacting coaches to remind their players to volunteer in this study, the response rate was 24% (74 out of 314 players) and must be interpreted as low [24]. This study followed a bottom-up approach and, therefore, it was totally dependent on the willingness of coaches and athletes to volunteer in the survey. It can be argued that a top-down approach, involving the official support of the Swiss Ice Hockey Federation, might have yielded a higher response rate.

Despite all efforts to evaluate a priori the feasibility and comprehensibility of the (translated) questionnaires by experts, one questionnaire was submitted incompletely. Because of the anonymity of the survey, it was impossible to contact respondents for clarification. Therefore, this dataset was deleted from the analysis.

A total of 62% of the 73 Swiss U20 ice hockey players volunteering in this study recorded injuries during the 2019/2020 season. Thirty-two players sustained one injury, 12 players reported two injuries, and one player suffered three injuries.

The survey assessed the extent of practice and games in this sample of 73 Swiss U20 ice hockey players. On average, volunteering athletes showed a training volume of 599 ± 140 h per year. The annual competition volume was 138 ± 37 h. Brunner et al. reported a high training load of 14 h per week (h/wk) of dryland training during the preparatory phase vs. 3 h/wk during the season [25]. The off-season phase from April to August in the present study amounted to 9 h/wk. Compared to second-division amateur ice hockey players [2], the Swiss U20 ice hockey players participating in this present study had a 66% higher training volume per year (204 ± 106 h) and a 37% higher competition volume (87 ± 58 h). The Swiss U20 Elite League aims to provide performance-oriented training and development of players in the context of national and international competitions. The focus is on training progression to achieve the highest level of performance, which automatically leads to growing personal commitment and increased training volume [26]. While a high volume of practice and games is needed to achieve high performance levels, it is known that a higher volume of training and competition may lead to overuse injuries in Swiss professional male ice hockey players [25]. Thus, this high load of practice and games may be a risk factor for injury in junior elite ice hockey players. Officials of junior teams should consider this when planning injury prevention measures.

The survey also tried to evaluate the incidence of injury among Swiss U20 ice hockey players during the 2019/2020 season. Based on the data from this sample under investigation, the IR was 0.66 per 1000 practice hours and 2.98 per 1000 game hours, resulting in an IRR of 4.5. This finding that injury risk is much higher in-game as compared to practice is corroborated by other studies. For example, Taeymans et al. [2] demonstrated an IRR in second-division amateur ice hockey players of 6.35, and Agel et al. [18] showed an IRR of 8.3 for division II college ice hockey players. Szukics et al. also indicated that players were more likely to get injured during games compared with training [27]. The intensity during ice hockey practice may be lower and involve more exercises than in game situations, which could explain the risk for injury differences between practice and games [18]. Regardless of level, injuries are more common during a game due to the higher speed of play and more frequent body checks compared to training, when parts of the game are often dedicated to specific training and building attacking and defensive systems. Players may be less inclined to check out teammates during practice and drills than their opponents during a game [27]. The fact that injury risk is much lower during practice as compared to games should also be recognized by officials of junior ice hockey teams when planning injury prevention measures.

The survey also elaborated on the context in which Swiss U20 ice hockey players sustained injuries. In this sample of 73 volunteers, 24% of all competition injuries occurred in the first third and 38% each in the second and third thirds of an ice hockey game. According to the studies by Tuominen et al. [19] and Agel et al. [18], the incidence of injury in the last two thirds was 34% and 36%, respectively. Player fatigue is also a risk factor for injury. Verschueren et al. [28] were able to demonstrate in a systematic literature review that acute fatigue is an intrinsic risk factor for lower limb injuries. In this context, fatigue can be seen as a complex phenomenon that manifests itself at both central and peripheral levels with continuous signal processing, feedback, and feedforward loops [28]. Smith et al. [29] assessed the frequency of injuries in 86 male high school ice hockey players as well as the influence of physical, situational, and psychosocial factors to determine predictors of injury. They conducted a multivariate analysis and found high fatigue and low vitality as risk factors for injury [29]. Fatigue can be caused by low aerobic or anaerobic endurance, but also by overtraining, depressive moods, and/or stress [29]. Thus, fatigue may be another risk factor for injury in elite junior ice hockey players.

This present study found that most injuries occurred during a body check (36%). Other injuries were caused by contact with a stick, puck, or skate (22%), an accidental collision (9%) with an opponent player, or a fist fight (2%). In 31% of cases, injuries occurred without opponent contact or had other causes. Of those, 30% involved the boards and 56% of injuries resulted from contact with another player. These findings corroborate the existing literature. For example, Agel et al. [18] indicated that contact with another player caused most injuries (47.7%), while Tuominen et al. [19] described a similarly high proportion of injuries (32%) resulting from body checks. In 13% of cases, the reason for an injury was contact with a stick or the puck. According to this present study and the literature, most injuries result from body contact such as checks or collisions. Therefore, fair play and tougher penalties can be good approaches for prevention. Another aspect which receives less attention is that most studies allocate a single mechanism of injury to each of the injuries recorded. Mölsä et al. [30] pointed out that injuries arise from a combination of mechanisms, and it seems relatively challenging to filter out the “most important” injury mechanism.

Muscle and tendon injuries (20%), contusions and sprains (18%), concussion (18%), and fractures (10%) were dominant injuries observed in the participants of this study. Skin injuries as well as joint injuries and ligament lesions each accounted for 7%. Azuelos et al. [31] found a distribution of injury type into distortions (21.7%), contusions (21.3%), skin injuries (15.1%), and muscle strains (13.2%) in ice hockey players. Tuominen et al. [19] listed skin injuries (24%) as the most common injury type, followed by contusions (22%), sprains (18%), concussions (10%), and fractures (10%) in junior ice hockey players. The higher injury rate can be explained by the change in the characteristics of the game (e.g., speed) as well as a higher proportion of physical contact between players, who are on average heavier, bigger, and stronger than in the past [31]. Contusions, for example, decreased by about 10% in the period from 1961 to 2000, while joint sprains decreased by almost 9% and muscle strains increased by almost 12% over time [31].

In this present sample of elite junior ice hockey athletes, concussions were reported in 24% of the injuries occurring during practice (50%) and games (43%). Concussions were caused in 71% of cases by contact with opponents or the playing equipment (57% body check and 7% each by a puck or stick). Åman et al. [11] reported that professional ice hockey players had the highest incidence of injury to the head and neck as a result of aggressive body checking. Tuominen et al. [19] attributed 10% of all injuries to concussions. Checks to the head (48%) and bodychecks (23%) were the most common causes. Contact with another player was also the most common cause (60.2%) of concussion [18].

The findings of this study are comparable to those from North America and the international championships. In under-20 World Championships ice hockey players, the most common anatomical region was the head and face [19]. Emery et al. [5] demonstrated a significantly increased overall risk of injury and a threefold increased risk of concussion in PeeWee players who played in a league where bodychecking was allowed in games compared to a league where bodychecking was not allowed. In college hockey, the injury rate per players at practice was 2.2 injuries per 1000 exposure hours in training and 13.8 injuries per 1000 exposure hours in competition [32]. These results are higher than those in the present study. A major reason for this difference is that the ice rinks are smaller in North America, resulting in more battles. In Swiss professional male ice hockey players, muscle strains and concussions were the most frequent time-loss injuries. It is known that player-to-player contact is the most frequent mechanism of injury [26]. The size of the ice rink and the speed are factors for injuries. A high amount of injuries sustained in practice are due to noncontact mechanisms [18].

In this study, time loss was used as a proxy for injury severity. This study found that in 58% of the reported injuries, the player was absent for less than four weeks (minor injury), while in 42% of the cases, the player was absent for four weeks or more (severe injury). According to Tuominen et al. [19], 59% of players were back in training after an injury period lasting up to one week. This corresponds to the definition of a “serious injury”. Ten per cent of the injured players had to take a break of at least three weeks.

Different diagnostic definitions of injury severity may partly explain differences in the data from different studies. In comparison to the study by Tuominen et al. [19], the present study did not include injuries that led to an interruption of the game but did not result in an interruption of the following training or competition. The data of the present study are consistent with those of the study by Taeymans et al. [2]. The latter reported an absence of more than four weeks (severe injury) in 39% of cases and an absence of one or two to three weeks in 25% of cases.

### Study Limitations

The present study is mainly descriptive without analyses of participant characteristics such as age, body composition, and physical performance in association with sports-related injuries during one season. Therefore, factors of body composition and physical (motor) performance of ice hockey players that may influence the rate, locations, and severity of the sports-related injuries were not evaluated.

During a retrospective questionnaire survey, recall bias may hamper the study results. To avoid this limitation, future studies should collect injury data prospectively and over several seasons.

Another limitation of this study is the sampling of approximately 24% of invited players. This led to potential detection and sampling bias. Future studies could address this limitation by increasing the total number of ice hockey players with face-to-face appointments and offering to be available for questions via Zoom or MS Teams. Also, a top-down approach with support of the Swiss Ice Hockey Federation may be considered to improve the response rate.

Furthermore, the data were collected from youth ice hockey players and, therefore, it was not possible to measure the extent to which injuries were not reported. The reporting threshold may differ between Swiss U20 ice hockey players. The question on the exposure period can also be made simpler. The completion of hours per week in the months of April to March sometimes resulted in a lack of understanding and was often filled in with the number of hours per month. This can lead to calculation errors. Here, the amount of competitive playing time could be simplified by specifying hours per month.

A further limitation is that some teams have a better staff base than others. This could lead to some injuries in teams without a team physiotherapist/physician resulting in injuries being documented and misclassified by athletes.

## 5. Conclusions

Of the 73 participating elite junior ice hockey athletes, 45 (62%) reported 1 to 3 injuries (totaling 59 injuries) during this period under evaluation. An injury rate per player of 0.66 injuries per 1000 exposure hours in practice and 2.98 injuries per 1000 exposure hours in games was found. The most common injury location was the head/neck region, while muscle and tendon injuries (20%) were documented as the most common type of injury. The time loss, a proxy of injury severity, was more than four weeks in 42% of the injuries (e.g., “severe injury”). This information can be used by physicians and physiotherapists for diagnosis or planning treatment modalities for junior elite ice hockey players who sustained an injury and by officials of junior ice hockey teams to develop injury prevention strategies.

## Figures and Tables

**Table 1:** Means and standard deviations of the anthropometric characteristics of the 73 Swiss U20 ice hockey players included in this study stratified for player position.

Player’s Position	Weight (kg)	Height (cm)	BMI (kg/m^2^)
Center (n = 11)	72.6 (±4.2)	179.0 (±3.7)	22.7 (±1.5)
Winger (n = 28)	78.4 (±6.5)	179.0 (±5.7)	24.4 (±1.8)
Defender (n = 26)	76.7 (±7.4)	178.0 (±5.3)	24.1 (±1.8)
Goalkeeper (n = 8)	75.3 (±6.4)	181.0 (±4,4)	23.2 (±2.4)

**Table 2:** Mean and standard deviations of athletic exposure [h] in practice and game stratified for off-season and peak season.

Exposure	Off-Season [h] (April 2019–August 2019)	Peak Season [h] (September 2019–March 2020)	Total [h]
Practice	223.0 ± 52.1	376.0 ± 105.0	599.0 ± 140
Game	16.5 ± 16.5	121.0 ± 33.8	138.0 ± 37.4
Practice and game	240.0 ± 55.3	497.0 ± 112	737.0 ± 144.0

**Table 3:** Type of injury in relation to the severity of the injury in the 45 injured Swiss U20 ice hockey athletes (season 2019/2020) out of the 73 athletes volunteering in this study.

Part of the Body	<1 Week	1–4 Weeks	>4 Weeks
Contusion/sprain	5	5	4
Ligament lesion	0	2	3
Muscle-tendon injury	3	6	6
Skin injury	4	1	0
Joint injury	0	0	5
Fracture	0	1	7
Concussion	0	10	4
Inflammation	3	4	2
Other	0	0	1
Total	15	29	32

**Table 4:** Injury mechanism in the 45 injured Swiss U20 ice hockey athletes (season 2019/2020) out of the 73 athletes volunteering in this study.

Injury Mechanism	% of Injuries
Contact with an opponent (unintended)	56%
Body check	38% and partly localized to the head (n = 8)
Contact with stick or puck	12%
Boarding	30%
Without external impact	12%

## Data Availability

The data presented in this study are available on request from the corresponding author.
